# Clinical Efficacy and Safety of Early Intravenous Administration of Beta-Blockers in Patients Suffering from Acute ST-Segment Elevation Myocardial Infarction Without Heart Failure Undergoing Primary Percutaneous Coronary Intervention: A Study-Level Meta-Analysis of Randomized Clinical Trials

**DOI:** 10.1007/s10557-023-07448-x

**Published:** 2023-04-01

**Authors:** Bing Sun, Chi Yao Wang, Rui Rui Chen

**Affiliations:** Department of Cardiology, Tang Du Hospital, Air Force Medical University, Shaanxi, China

**Keywords:** Acute ST-segment elevation myocardial infarction, primary percutaneous coronary intervention, intravenous beta-blockers

## Abstract

**Background:**

Several clinical studies have produced diverse results regarding the efficacy and safety of early intravenous beta-blockers in patients with acute ST-segment elevation myocardial infarction (STEMI). A study-level meta-analysis of randomized clinical trials (RCTs) comparing early intravenous beta-blockers versus placebo or routine care in STEMI patients undergoing primary percutaneous coronary intervention (PCI) was performed.

**Methods:**

A database search was conducted using PubMed, EMBASE, the Cochrane Library, and Clinicaltrials.gov for randomized clinical trials (RCTs) that compared intravenous beta-blockers versus placebo or routine care in STEMI patients who underwent primary PCI. The efficacy outcomes were infarct size (IS, % of LV) and the myocardial salvage index (MSI) based on magnetic resonance imaging, electrocardiographic findings, heart rate, ST-segment reduction percent (STR%), and complete STR. Safety outcomes included arrhythmias in the first 24 h (ventricular tachycardia and fibrillation [VT/VF], atrial fibrillation [AF], bradycardia, and advanced atrioventricular [AV] block), cardiogenic shock and hypotension during hospitalization, left ventricular ejection fraction (LVEF), and major adverse cardiovascular events (cardiac death, stroke, reinfarction, and heart failure readmission) at follow-up.

**Results:**

Seven RCTs with 1428 patients were included in this study, with 709 patients in the intravenous beta-blockers and 719 in the control group. Intravenous beta-blockers improved MSI compared to the control group (weighted mean difference [WMD] 8.46, 95% confidence interval [CI] 3.12–13.80, *P* = 0.002, I^2^ = 0%), but no differences were observed in IS (% of LV) between groups. Compared to the control group, the intravenous beta-blockers group had a lower risk of VT/VF (relative risk [RR] 0.65, 95% CI 0.45–0.94, *P* = 0.02, I^2^ = 35%) without an increase of AF, bradycardia, and AV-block and significantly decreased HR, hypotension. LVEF at 1 week ± 7 days (WMD 2.06, 95% CI 0.25–3.88, *P* = 0.03, I^2^ = 12%) and 6 months ± 7 days (WMD 3.24, 95% CI 1.54–4.95, *P* = 0.0002, I^2^ = 0%) was improved in the intravenous beta-blockers group compared to the control group. Subgroup analysis showed that intravenous beta-blockers before PCI decreased the risk of VT/VF and improved LVEF compared to the control group. Furthermore, sensitivity analysis showed that patients with a left anterior descending (LAD) artery lesion had a smaller IS (% of LV) in the intravenous beta-blockers group compared to the control group.

**Conclusion:**

Intravenous beta-blockers improved the MSI, decreased the risk of VT/VF in the first 24 h, and were associated with increased LVEF at 1 week and 6 months following PCI. In particular, intravenous beta-blockers started before PCI is beneficial for patients with LAD lesions.

## Introduction

Acute ST-segment elevation myocardial infarction (STEMI) is the most common cause of death in patients with cardiovascular disease [1]. Primary percutaneous coronary intervention (PCI) has emerged as the mainstay treatment for STEMI [2]. Although instantaneous ischemia-reperfusion may cause myocardial cell death, coronary microvascular obstruction, and malignant arrhythmia [3], timely reperfusion can reduce the extent of myocardial infarction (MI) [4].

In a previous study, it was shown that intravenous beta-blockers for acute MI did not improve outcomes when combined with thrombolytic therapy [5]. For hemodynamically stable patients undergoing primary PCI, guidelines recommend using intravenous beta-blockers (class IIa) [6]. However, multiple randomized controlled trials (RCTs) have shown conflicting results regarding the efficacy and safety of intravenous beta-blockers before primary PCI for STEMI [7–13]. The extent of the size of the myocardial infarct after STEMI is a well-known predictor of adverse outcomes, including mortality [14]. The METOCARD-CNIC (Effect of Metoprolol in Cardio-protection During an Acute Myocardial Infarction) trial demonstrated that intravenous metoprolol before PCI reduced the infarct size based on cardiac magnetic resonance imaging (MRI) [9]. However, the EARLY-BAMI (Early Beta-blocker Administration before reperfusion primary PCI in patients with ST-elevation Myocardial Infarction) trial did not show any effect of intravenous metoprolol before primary PCI on infarct size based on MRI [11]. Miyamoto et al. showed that intravenous landiolol increased the myocardial salvage index (MSI) on cardiac MRI but infarct size was not significantly reduced [13]. In other studies, peak creatine kinase (CK), CK isoenzyme-MB (CK-MB), and cardiac troponin I/T (cTnI/T) were used to assess myocardial infarct size as the primary outcome [7, 10, 12]. Additionally, using beta-blockers in the hyperacute phase of STEMI may predispose to bradycardia, heart block, cardiogenic shock, or heart failure due to the negative inotropic and chronotropic effects of beta-blockers.

The meta-analysis that was published in 2013 included studies before 2011 using intravenous beta-blockers that have shown positive clinical efficacy [15]. This included patients suffering from acute coronary syndrome (ACS) or STEMI with or without heart failure, while in our study, only patients were included who suffered from STEMI with Killip I or II. The purpose of this study was to investigate the efficacy and safety profile of intravenous beta-blockers in patients with acute STEMI undergoing PCI in the current era of primary PCI.

## Methods

This systematic review and meta-analysis were performed according to the PRISMA 2020 statement guideline [16].

### Inclusion and Exclusion Criteria

Inclusion criteria: Patients included presented with STEMI within 12 h of symptom onset to reperfusion and a Killip class I or II. All included trials were randomized controlled trials (RCTs), with or without blind trials. The criteria for diagnosis of STEMI included in the study were in line with the guidelines issued by the American College of Cardiology/American Heart Association (ACC/AHA) and European Society of Cardiology (ESC) [4, 6]. Exclusion criteria were as follows: Patients with a low systolic blood pressure <90 mmHg or mean arterial pressure <65 mmHg, heart rate (HR) <60 beats/min, and AV block type II or III. Animal experiments, case reports, reviews, meta-analyses, conference proceedings without a full manuscript and non-RCTs were excluded.

### Intervention Measures and Outcomes

Intravenous beta-blockers group: intravenous administration of beta-blockers; control group: placebo or routine care. The efficacy outcomes were IS (% of LV) and MSI% based on magnetic resonance imaging (MRI) and electrocardiographic (ECG) results (heart rate (HR), percentage of ST segment reduction (STR%), and complete STR). The safety outcomes were arrhythmias in the first 24 h (ventricular tachycardia and ventricular fibrillation (VT/VF), atrial fibrillation (AF), bradycardia, advanced atrioventricular block (AV-block)), cardiogenic shock and hypotension during hospitalization, left ventricular ejection fraction (LVEF), and major adverse cardiovascular events (MACEs (cardiac death, stroke, reinfarction, and heart failure) at follow-up.

### Search Strategy

Databases including PubMed, Web of science, Embase, the Cochrane Library, and Chinese biomedical literature database (CBM) were systematically searched from inception to December 1, 2022, for RCTs examining the use of intravenous beta-blockers in the acute phase of STEMI. A combination of Medical Subject Heading and EMTREE terms were used, including “beta-blockers,” “adrenergic beta-antagonists”, “STEMI,” “metoprolol,” “esmolol,” “landiolol,” and relevant articles included in references were traced.

### Study Selection and Data Collection

Two investigators independently screened the titles and abstracts of articles identified in the electronic search. The full text of potentially eligible articles was retrieved and reviewed to determine if the pre-specified inclusion criteria were met. Moreover, a third investigator was consulted when needed. Articles were published in English or Chinese, and contained data from an RCT comparing intravenous beta-blockers versus control or routine care in STEMI patients who underwent PCI. Observational studies, case reports, case series, abstracts, conference proceedings, reviews, and letters to the editor were excluded from the study. Extracted data contained the following: author and publication year; sample size; patient demographics; cardiovascular risk stratification (BMI, hypertension, diabetes mellitus, dyslipidemia, smoking history); number of stenosed vessels; ischemia duration; Killip Class; infarct related artery; perioperative medication; study characteristics included study design, intervention methods, outcomes, and follow-up time. The Cochrane quality assessment tool was used in RCTs [17].

### Statistical Analysis

RevMan5.4 statistical software was used for meta-analysis. The heterogeneity of included articles was analyzed using the X2 test (test level a = 0.1) and evaluated using I^2^ statistics. If the heterogeneity test results were I^2^ < 50%, the fixed effect model was used for meta-analysis. If the consistency test results were I^2^ ≥ 50%, the random effect model was used for meta-analysis. The relative risk (RR) represented the effect index when the outcome index was a dichotomous variable, whereas the WMD was used for continuous variables. There were only 7 studies < 10, therefore, there was no need to carry out the test of publication bias. Data were considered statistically significant when *P* < 0.05.

## Results

### Screening of Literature

**Fig. 1:**
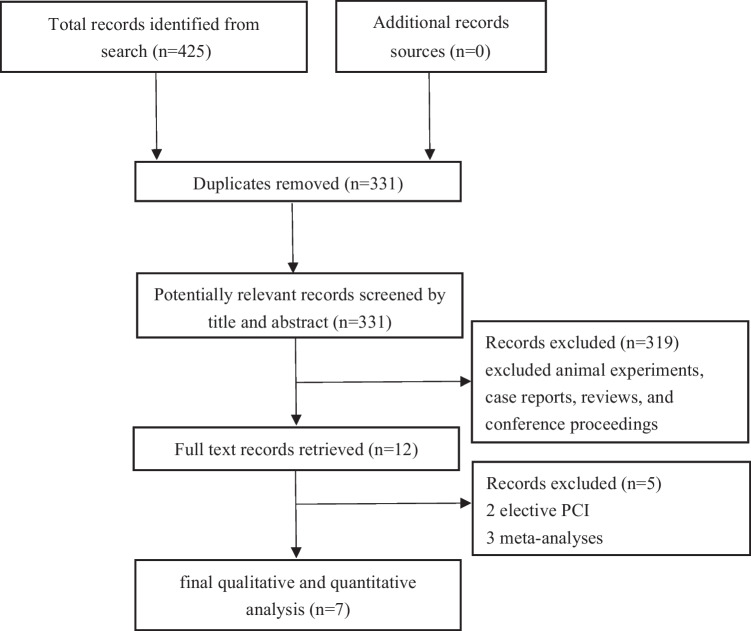
PRISMA flow diagram

**Fig. 2:**
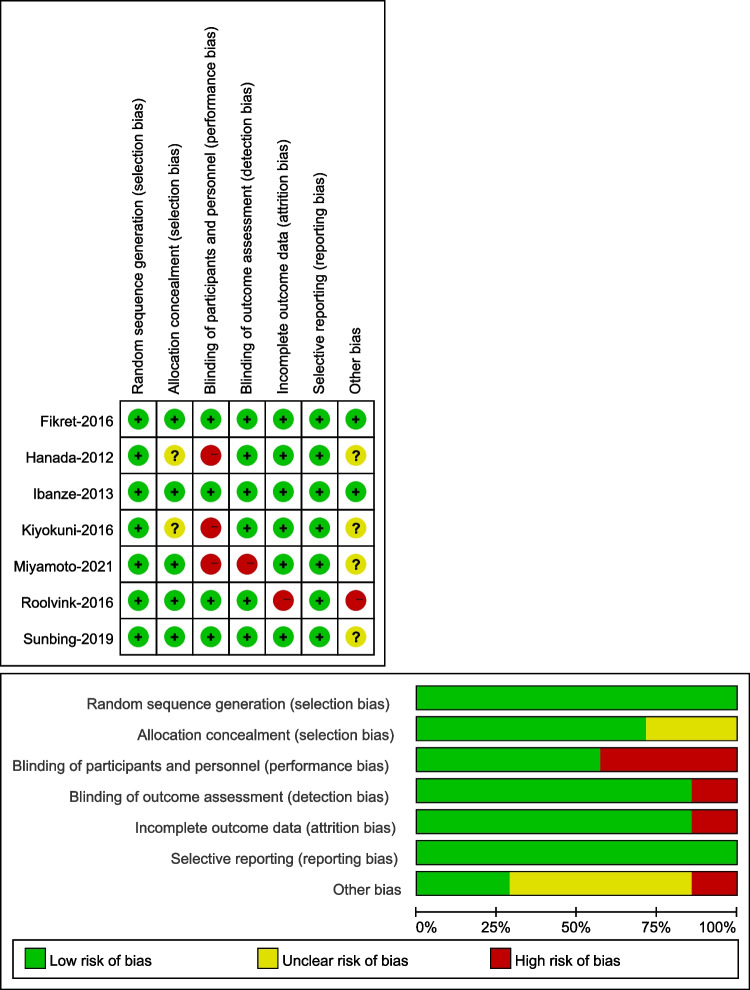
Cochrane quality assessment

A total of 425 studies were retrieved from PubMed, EMBASE, the Cochrane Library, and CBM, and 94 articles were selected after the removal of duplicate papers. After evaluating the titles and abstracts (including reviews, animal experiments, case reports, and conference proceedings), 319 articles and 12 articles were excluded after reading the full text. Two trials that included patients elective PCI [18, 19] and three meta-analyses were excluded [20–22]. Finally, seven articles were included in the qualitative and quantitative studies (Fig. 1). The results of Cochrane quality assessment are presented in Fig. 2.

### Study Characteristics

All included RCTs used a selective beta-1 receptor antagonist and treated STEMI patients with PCI. Intravenous metoprolol was used in the study performed by Roolvink et al. [11] (two doses of 5 mg, including a first dose in the ambulance, and second dose just before PCI) and Ibanze et al. [9] (three doses of 5 mg, interval 2 min, prior to PCI). A loading dose followed by a 24-h, continuous, weight-based esmolol infusion was carried out in the study performed by Er et al. [10], targeting an HR of 60 beats per min immediately (within 60 min) after primary PCI. Bing et al. treated with a loading dose before PCI followed by continuous, weight-based esmolol during PCI until PCI was completed [7]. Patients with symptoms lasting <4.5, 6 or 12 h were included in the study. Hanada et al. started treatment at 3 μg/kg per min, 24-h, continuous landiolol infusion immediately after primary PCI [8]. In the study by Kiyokuni et al. [12], an intravenous infusion of landiolol was given just before reperfusion at 3 μg/kg per min, and this dose was continued within 6–12 h after the primary PCI. Intravenous infusion of landiolol 3 μg/kg per min before PCI and continued to a total of 50 mg was used in the study by Miyamoto et al. [13]. Other study characteristics are presented in Table 1. Baseline and procedural characteristics between two groups are presented in Table 2.

**Table 1:** Study characteristics

Author and year	Design	Sample size		Intervention	Outcomes	Follow-up time
		Experimental	Control			
Hanada et al. 2012	RCT	47	49	Continuous administration of landiolol a 3 μg/kg per min for 24h after PCI	Heart rate, arrhythmias in the first 24 h MACEs at 6 months, LVEF at 2 weeks and 6 months	In-hospital, 6 months
Ibanze et al. 2013	RCT	131	139	Three 5 mg, interval 2 min metoprolol, before PCI	IS on MR, peak and AUC CK, LVEF at 7 days and 6 months, Arrhythmias in the first 24h, ECG before reperfusion	In-hospital, 7 days,12months, 5-year
Roolvink et al. 2016	RCT	336	346	First dose 5 mg in ambulance, Second dose 5mg metoprolol at immediately before PCI	IS on MR at 30 days, peak CK, CK-MB, cTnI, AUC CK at 24h, MACEs at 30days, Arrhythmias in the first 24h, ECG after PCI, LVEF at 1month	In-hospital, 30 days, 1-year
Kiyokuni et al. 2016	RCT	55	60	3 μg/kg per min landiolol was continued within 6–12 h after the primary PCI	Heart rate and ECG after PCI, peak CK, MACEs at 12 months, Arrhythmias in the first 24h,	In-hospital, 12 months
Er et al. 2016	RCT	50	50	A loading dose esmolol followed by weight-based 24h immediately after PCI	Heart rate, Peak and AUC CK, CK-MB, cTnI/T, LVEF at 1 month and 6 months, MACEs at 6 months	In-hospital, 6 months
Bing et al. 2019	RCT	58	59	A loading dose before PCI and continuous, weight-based esmolol during PCI	Heart rate and ECG after PCI, peak CK-MB, cTnI, Arrhythmias in the first 24 h, LVEF at 1 week, 1 month, 3 months, 6 months, MACEs at 6 months	In-hospital, 6 months
Miyamoto et al. 2021	RCT	23	24	3 μg/kg per min landiolol was started before PCI and continued to a total of 50 mg	IS on MR at 5-7days, peak CK and CK-MB, Arrhythmias in the first 24 h	In-hospital

**Table 2:** Baseline characteristics 1 and procedural characteristics

Author and year	Group	Age		Male	BMI (kg/m^2^)	Hypertension	Diabetes (mmol/L)	Hyperlipemia (mmol/L)	Smoking
Hanada et al. 2012	Landiolol	63.2±1.6		40 (80)	25.3±0.6	33(70)	20(43)	38(81)	28 (60)
	Control	61.5±1.9		40 (85)	24.9±0.5	35(71)	21(43)	38(78)	26 (53)
Ibanze et al. 2013	Metoprolol	58.7±12.7		119 (85.6)	27.6±3.7	50(40.3)	31(23.3)	53(39.8)	71 (53)
	Control	58.2±10.8		114 (87)	27.9±3.9	54(40.2)	24(18.8)	51(40.2)	69 (53.9)
Roolvink et al. 2016	Metoprolol	62.39±12.42		84 (75)	27.11±4.45	135(40.3)	48(14.3)	—	—
	Control	62.46±12.58		88 (74.6)	27.40±4.11	133(38.7)	62(17.9)	—	—
Kiyokuni et al. 2016	Landiolol	65±13		49 (81)	24.2±3.7	30(50)	19(32)	38(63)	34 (57)
	Control	65±13		43 (78)	23.3±3.3	35(64)	9(20)	36(65)	22 (40)
Er et al. 2016	Esmolol	57.9±11.2		41 (82)	26.6±3.8	27(54)	6(12)	13(26)	30 (62)
	Control	61.4±12.2		36 (72)	26.1±4.1	27(54)	6(12)	16(32)	22 (44)
Bing et al. 2019	Esmolol	55±11		50 (85)	26.6±3.2	30(51)	44(75)	30(51)	44 (75)
	Control	55±10		49 (84)	26.5±5.2	28(48)	45(78)	24(41)	39 (67)
Miyamoto et al. 2021	Landiolol	64±11		21 (91)	23.6±2.8	15(65)	7(30)	9(39)	16 (69)
	Control	67±13		21 (87)	23.9±3.4	15(62)	6(25)	8(33)	9 (37)
Killip I	Ischemia duration	NO. of stenotic vessels			Infarct related artery			Perioperative medication	
		1	2	3	LAD	LCX	RCA	Beta-blocker	ACEI or ARB
46 (98)	382.5±43.7	24 (51)	8 (17)	15 (32)	25 (54)	11 (23)	6 (12)	45 (96)	46 (98)
45 (92)	353.7±35.1	26 (53)	10 (20)	13 (27)	29 (59)	11 (23)	14 (29)	46 (94)	46 (94)
128 (92.5)	197±61	NR	NR	NR	139	NR	NR	NR	NR
114 (87)	187±66	NR	NR	NR	131	NR	NR	NR	NR
NR	195.5±262.5	175 (53)	100 (30.3)	39 (11.8)	154	NR	NR	260 (78.1)	229 (68.8)
NR	201.6±262.1	201 (59.3)	71 (20.9)	46 (13.6)	166	NR	NR	249 (73)	232 (68)
NR	244±167	NR	NR	NR	31	NR	NR	NR	NR
NR	243±172	NR	NR	NR	25	NR	NR	NR	NR
NR	NR	NR	NR	NR	17 (34)	5(10)	28 (56)	NR	NR
NR	NR	NR	NR	NR	26 (53.1)	5(10)	18 (36.7)	NR	NR
30 (51)	426±180	26 (43)	≥2	33 (57)	40 (68)	6 (10)	13 (22)	47 (81)	38 (65)
32 (55)	414±210	25 (44)	≥2	33 (56)	38 (66)	3 (5)	17 (29)	52 (88)	33 (56)
23 (100)	219±101	NR		NR	21 (91)	0	2 (9)	NR	NR
24 (100)	273±140	NR		NR	21 (88)	1 (4)	2 (38)	NR	NR

### Efficacy Outcomes

#### Infarct Size (IS, % of LV) and Myocardial Salvage Index (MSI)

**Fig. 3:**
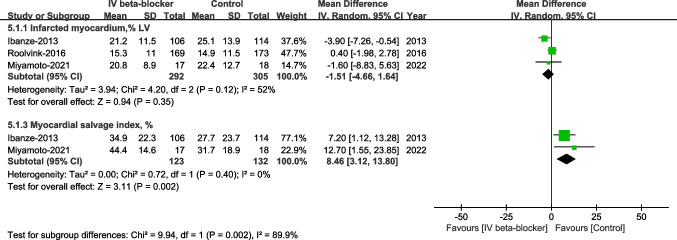
Infarcted myocardium (%LV) and myocardial salvage index, %

No significant differences in proportions of IS (% of LV) (WMD –1.51, 95% CI –4.66 to 1.64, *P* = 0.35, I^2^ = 52%). The MSI in the intravenous beta-blockers group was significantly greater than in the control group (WMD 8.46, 95% CI 3.12-13.80, *P* = 0.002, I^2^ = 0%, Fig. 3).

#### ECG Results

**Fig. 4:**
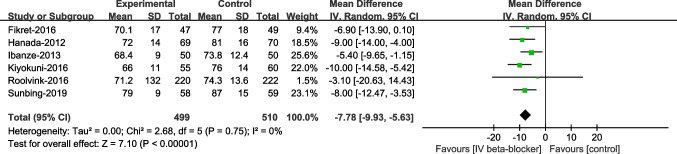
Mean heart rate (HR)

**Fig. 5:**
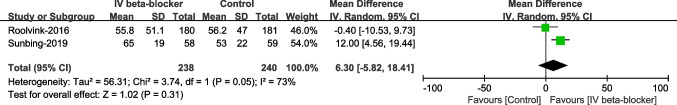
Percentage of ST-segment reduction (STR%)

**Fig. 6:**
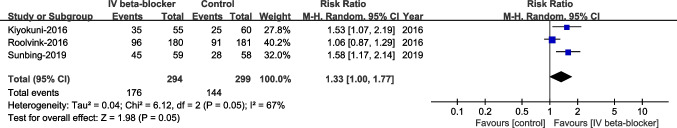
Complete ST-segment reduction (STR)

HR was significantly lower in the intravenous beta-blockers group compared to the control group (WMD –7.78, 95% CI –9.93 to –5.63, *P* < 0.00001, I^2^ = 0%). No significant differences were observed in STR% (WMD 6.30, 95% CI –5.82-18.41, *P* = 0.31, I^2^ = 73%), but the number of complete STR was significantly higher in the intravenous beta-blockers group compared to the control group (RR 1.33, 95% CI 1.00–1.77, *P* = 0.05, I^2^ = 67%, Figs. 4, 5, and 6).

### Safety Outcomes

#### Arrhythmias in the First 24 h After PCI

**Fig. 7:**
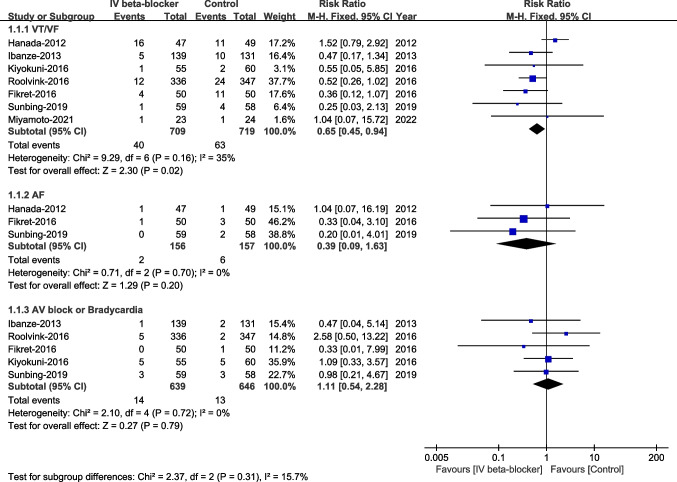
Arrhythmias in the first 24 h after PCI

VT/VF was significantly lower in the intravenous beta-blockers group compared to the control group (RR 0.65, 95% CI 0.45–0.94, *P* = 0.02, I^2^ = 35%). Moreover, no significant differences were observed in AF (RR 0.39, 95% CI 0.09–1.63, *P* = 0.20, I^2^ = 0%) and advanced AV block/bradycardia (RR 1.11, 95% CI 0.54–2.28, *P* = 0.79, I^2^ = 0%) between the two groups, Fig. 7.

#### Cardiogenic Shock and Hypotension at Hospitalization

**Fig. 8:**
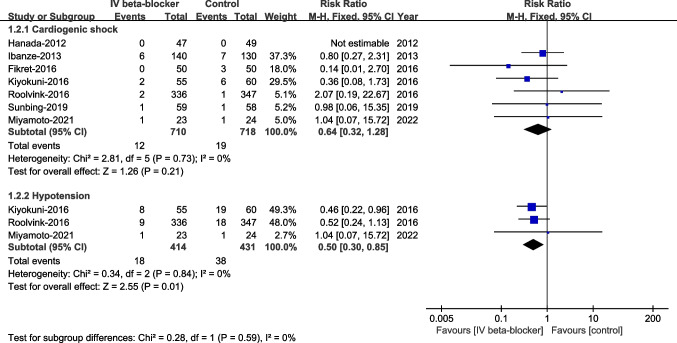
Cardiogenic shock and hypotension at hospitalization

There were no significant differences in cardiogenic shock (RR 0.64, 95% CI 0.32–1.28, *P* = 0.21, I^2^ = 0%), but hypotension was significantly lower in the intravenous beta-blockers group compared to the control group (RR 0.50, 95% CI 0.30–0.85, *P* = 0.01, I^2^ = 0%, Fig. 8).

#### Left Ventricular Ejection Fraction (LVEF)

**Fig. 9:**
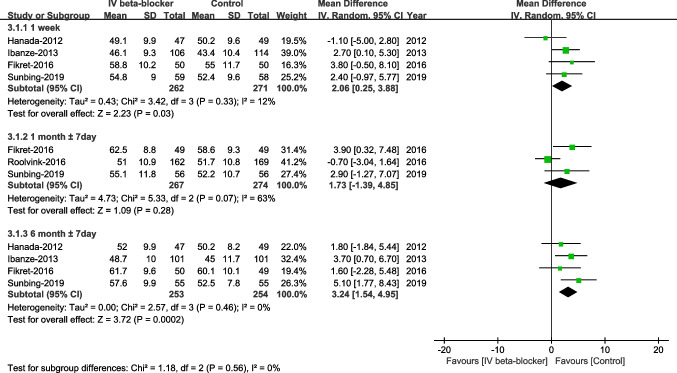
Left ventricular ejection fraction (LVEF)

No significant differences in LVEF were observed at 1 month ± 7days between the two groups (WMD –1.73, 95% CI –1.39-4.85, *P* = 0.28, I^2^ = 63%). The LVEF at 1 week ± 7 days (WMD 2.06, 95% CI 0.25–3.88, P = 0.03, I^2^ = 12%) and 6 months ± 7days (WMD 3.24, 95% CI 1.54–4.95, *P* = 0.0002, I^2^ = 0%) after PCI was significantly improved in patients treated with an early intravenous beta-blocker, Fig. 9).

#### MACEs (Cardiac Death, Stroke, Reinfarction, and Heart Failure) at Follow-Up Time

**Fig. 10:**
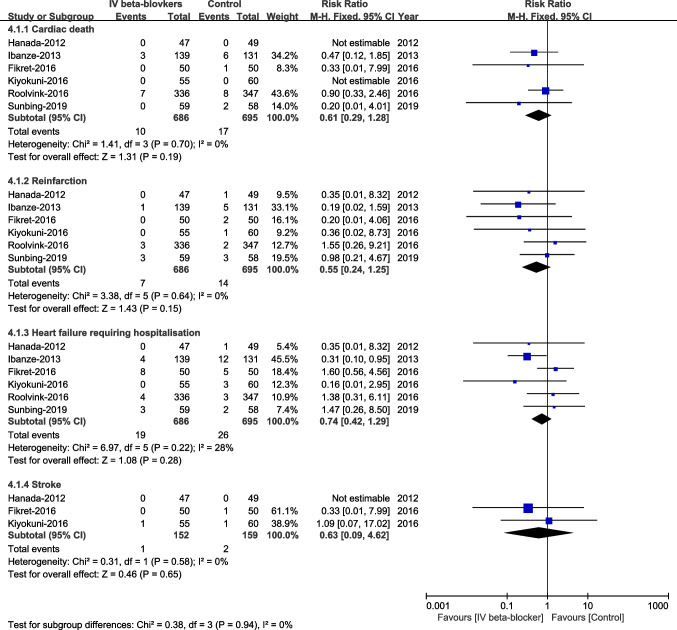
MACEs (cardiac death, stroke, reinfarction, and heart failure) at follow-up time

No significant differences were observed in cardiac death, stroke, reinfarction, and heart failure readmission between groups (Fig. 10).

#### Subgroup-Analysis

Subgroup-analysis was performed on outcomes (VT/VF, AV block/bradycardia, cardiogenic shock, heart failure readmission, and LVEF) according to different types of beta-blockers and intravenous beta-blockers were started before or after PCI. VT/VF was significantly lower in metoprolol (*P* = 0.02) and esmolol (*P* = 0.02) subgroups compared to control groups. LVEF at 1 week, 1 month, and 6 months were significantly lower in the esmolol subgroup compared to the control group. No differences were observed in AV block/bradycardia, cardiogenic shock, heart failure readmission in the subgroups (Table 3). VT/VF was significantly lower in the intravenous beta-blockers group compared to the control group (*P* = 0.009) in the before-PCI subgroup and was not significantly different between groups in the after-PCI subgroup (*P* = 0.70). The LVEF at 1 week and 6 months was significantly improved in the intravenous beta-blockers group compared to the control group (*P* = 0.01, *P* = 0.0001) in the before-PCI subgroup and was not significantly different between groups in the after-PCI subgroup (*P* = 0.61, *P* = 0.21), Table 4.

**Table 3:** Subgroup-analysis of different kinds of beta-blockers

Subgroup	Number of studies	RR/WMD (95%CI)	*P* value
VT/VF	7	0.65 [0.45, 0.94]	0.02
metoprolol	2	0.50 [0.28, 0.89]	0.02
esmolol	2	0.33 [0.13, 0.87]	0.02
landiolol	3	1.35 [0.73, 2.48]	0.34
AV block/bradycardia	5	1.11 [0.54, 2.28]	0.79
metoprolol	2	1.50 [0.43, 5.24]	0.52
esmolol	2	0.77 [0.20, 3.02]	0.70
landiolol	1	1.09 [0.33, 3.57]	0.89
Cardiogenic shock	7	0.64 [0.32, 1.28]	0.21
metoprolol	2	0.95 [0.36, 2.47]	0.91
esmolol	2	0.33 [0.05, 2.07]	0.24
landiolol	3	1.04 [0.07, 15.92]	0.98
Heart failure readmission	6	0.74 [0.42, 1.29]	0.28
metoprolol	2	0.52 [0.22, 1.20]	0.12
esmolol	2	1.56 [0.64, 3.84]	0.33
landiolol	2	0.21 [0.03, 1.81]	0.16
LVEF at 1 week	4	2.06 [0.25, 3.88]	0.03
esmolol	2	2.93 [0.28, 5.59]	0.03
LVEF at 1 month ± 1 week	3	1.73 [-1.39, 4.85]	0.28
esmolol	2	3.48 [0.76, 6.19]	0.01
LVEF at 6 months ± 1 week	4	3.24 [1.54, 4.95]	0.0002
esmolol	2	3.50 [0.08, 6.92]	0.04

*VT/VF*, Ventricular tachycardia and fibrillation

*LVEF*, Left ventricular ejection fraction

**Table 4:** Subgroup-analysis of intravenous beta-blockers before or after PCI

Subgroup	Number of studies	RR/WMD (95%CI)	*P* value
VT/VF	7	0.65 [0.45, 0.94]	0.02
Before PCI	4	0.49 [0.29, 0.84]	0.009
After PCI	3	0.90 [0.54, 1.52]	0.70
AV block/bradycardia	5	1.11 [0.54, 2.28]	0.79
Before PCI	3	1.28 [0.48, 3.38]	0.62
After PCI	2	0.91 [0.31, 2.70]	0.87
Cardiogenic shock	7	0.64 [0.32, 1.28]	0.21
Before PCI	4	0.96 [0.41, 2.26]	0.93
After PCI	3	0.28 [0.07, 1.09]	0.07
Heart failure readmission	6	0.74 [0.42, 1.29]	0.28
Before PCI	2	0.63 [0.30, 1.22]	0.62
After PCI	2	0.92 [0.39, 2.19]	0.85
LVEF at 1 week	4	2.06 [0.25, 3.88]	0.03
Before PCI	2	2.59 [0.53, 4.65]	0.01
After PCI	2	1.73 [-1.39, 4.85]	0.61
LVEF at 6 months±1 week	4	3.24 [1.54, 4.95]	0.0002
Before PCI	2	4.33 [2.10, 6.56]	0.0001
After PCI	2	1.71 [-0.95, 4.36]	0.21

*VT/VF*, Ventricular tachycardia and fibrillation

*LVEF*, Left ventricular ejection fraction

#### Sensitivity Analysis

**Fig. 11:**
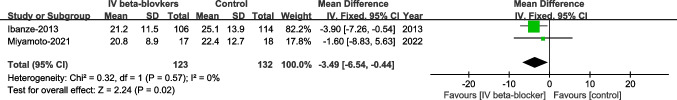
Sensitivity analysis of infarcted myocardium (%LV)

Sensitivity analysis was performed on IS (% of LV) and follow-up MACEs. When Roolvink et al. was excluded [10], the IS (% of LV) was significantly smaller in the intravenous beta-blockers group compared to the control group (Fig. 11).

## Discussion

The main findings of our study can be summarized as follows.

1. Intravenous beta-blockers improved the MSI, decreased HR, and were associated with a higher rate of complete STR.
2. Intravenous beta-blockers significantly decreased VT/VF and did not increase advanced AV block/bradycardia in the first 24 h after PCI. No significant differences were observed in cardiogenic shock between groups, but the rate of hypotension was significantly lower in the intravenous group compared to the control group at hospitalization.
3. Intravenous beta-blockers improved the LVEF at 1 week and 6 months.
4. Subgroup-analysis showed that intravenous beta-blockers in the before-PCI subgroup decreased the VT/VF and improved the LVEF at 1 week and 6 months, but did not show any differences between the two groups in the after-PCI subgroup.
5. Sensitivity analysis showed that patients with an LAD lesion were associated with a smaller IS (% of LV) in the intravenous beta-blockers group compared to the control group.

The results of our study showed that intravenous beta-blockers did not reduce the IS (% of LV). However, when the paper of Roolvink et al. was excluded [10], the IS (% of LV) was significantly lower in the intravenous group compared to the control group. Intravenous metoprolol was used in Ibance et al. and Roolvink et al., but the results were not consistent between the two studies. One possible explanation could be the dose of metoprolol. In Ibance et al. [9], the dose was three times 5 mg (15 mg target dose), compared to two times 5 mg (10 mg target dose) in the study by Roolvink et al. [10]. Another explanation could be that 18.8% of patients in the Roolvink et al. [10] trial were on long-term beta-blocker treatment before admission, whereas long-term oral beta-blocker treatment was an exclusion criterion in the Ibance et al. paper [9]. A third explanation could be that treatment with an early beta-blocker may only be beneficial in patients with an anterior infarction, and less beneficial or even harmful in patients with an inferior location. Ibance et al. and Miyamoto et al. included patients undergoing STEMI mainly with a left anterior descending (LAD) lesion (93%) [9, 13]. Intravenous beta-blocker improved the MSI and was associated with a smaller IS (% of LV) in the intravenous beta-blockers group compared to the control group in the meta-analysis of above two trials. Therefore, it is hypothesized that patients with an LAD lesion would benefit more from therapy with intravenous beta-blockers.

In the COMMIT trial [23], intravenous metoprolol did not improve survival in STEMI patients. However, this was mainly caused by a higher incidence of cardiogenic shock in patients treated with an early beta-blocker, possibly due to inclusion of patients with heart failure. In our included studies, patients with Killip III or IV were excluded. No significant differences were found in cardiogenic shock between the intravenous beta-blockers group (1.7%) and control group (2.8%). In addition, hypotension was significantly lower in the intravenous group (4.4%) compared to the control group (8.8%). LVEF at 1 week was improved in the intravenous beta-blockers group compared to in the control group. Taken together, these findings indicated that intravenous beta-blockers may improve left ventricular function better than the control group during hospitalization, which may be associated with a lower rate of hypotension in the intravenous beta-blocker group. Furthermore, the LVEF in the intravenous beta-blockers group was improved at 6 months compared to the control group. Microvascular obstruction (MVO) and intramyocardial hemorrhage (IMH) are independent predictors of adverse LV remodeling and clinical outcomes after STEMI [24, 25]. An improved LVEF may be due to decreased MVO and IMH in the intravenous group compared to the control group [26]. Five-year outcomes of METOCARD-CNIC showed lower MACEs and heart failure admissions in the intravenous group, which was not consistent with our results [27]. One potential reason was that the other six studies only had a short- and mid-term follow-up time, while early intravenous metoprolol had a long-term beneficial prognostic effect, particularly in patients with severely impaired LV systolic function. Intravenous metoprolol had more preserved global LV strain and infarct zone circumferential strain after STEMI which may contribute to lower MACEs and heart failure admissions during long-term follow-up [26].

In our study, the results of ECG showed that intravenous beta-blockers resulted in a lower HR, and higher complete STR after PCI. As mentioned above, bradycardia was similar between two groups. Therefore, compared to the control group, intravenous beta-blockers had effective and safe control of HR. A METOCARD-CNIC trial electrocardiographic study showed that the intravenous beta-blockers group also had a lower HR and total ST-segment elevation before reperfusion [28].

Subgroup-analysis showed that intravenous esmolol was associated with a lower risk of VT/VF compared to the control group and improved LVEF at 1 week, 1 month, and 6 months. Compared to two or three dose 5 mg of intravenous metoprolol, a loading dose followed by continuous intravenous esmolol better achieved the target HR and significantly faster recovery of HR and blood pressure (BP) in patients with STEMI [29]. Intravenous esmolol may be an effective alternative to intravenous metoprolol for patients with STEMI. Intravenous beta-blockers in the before-PCI subgroup were associated with a higher LVEF at 1 week and 6 months compared to the control group, while no significant differences were found between the two groups in the after-PCI subgroup. The results may indicate that the earlier intravenous beta-blockers were used, the more improved LV function.

Compared with a previous meta-analysis [20–22], the current report has included several updates. Meta-analysis of IS (% of LV), MSI, heart rate (HR), percentage of ST segment reduction (STR%), and complete STR was performed, which were not analyzed in previous studies. Additional subgroup and sensitivity analysis was also performed. According to our results, several new meaningful findings were found. First, intravenous beta-blockers improved the MSI%, decreased HR, and were associated with a higher rate of complete STR. Second, intravenous beta-blockers before PCI decreased VT/VF and improved the LVEF at 1 week and 6 months, but did not show any differences between the two groups after PCI. Third, patients with an LAD lesion were associated with a smaller IS (% of LV) and improved MSI in the intravenous beta-blockers group compared to the control group.

### Limitations

This study has the following limitations: (1) baseline data cannot be completely balanced as not all the data from all individuals was obtained, which could have affected our results; (2) Inclusion and exclusion criteria, intervention measures were not consistent in all included studies, which may provide an underlying source of clinical heterogeneity in the meta-analysis; (3) All included studies had a small sample size, whereas a large sample size and multicenter RCTs are needed.

## Conclusion

According to the results of our study, intravenous beta-blockers during the acute phase of ST-segment elevation myocardial infarction (STEMI) were effective and safe. Intravenous beta-blockers reduced VT/VF and did not increase advanced A-V block or bradycardia in the first 24 h after primary percutaneous coronary intervention (PCI). Intravenous beta-blockers improved the myocardial salvage index (MSI) and LVEF at 1 week and 6 months. They also decreased long-term major adverse cardiovascular events (MACEs) compared to the control group. Patients with an LAD lesion may benefit more from intravenous beta-blockers before PCI. With the contemporary background, it is warranted to fully understand the underlying mechanism(s) of action of intravenous beta-blockers, and large RCTs will be required to more precisely determine the role of intravenous beta-blockers in STEMI in the era of PCI.

## Data Availability

The original contributions presented in the study are included in the article/Supporting Information Material, further inquiries can be directed to the corresponding authors.
